# Motor cortex outputs evoked by long-duration microstimulation encode synergistic muscle activation patterns not controlled movement trajectories

**DOI:** 10.3389/fncom.2022.851485

**Published:** 2022-08-11

**Authors:** Charles Capaday

**Affiliations:** ^1^Brain and Movement Laboratory, Department of Bioengineering, McGill University, Montreal, QC, Canada; ^2^Department of Health and Human Physiology, The University of Iowa, Iowa City, IA, United States

**Keywords:** motor cortex, muscle map, movement trajectory, intra-cortical microstimulation (ICMS), cortical circuits, muscle synergies, bicuculline

## Abstract

The effects of intracortical microstimulation (ICMS) parameters on the evoked electromyographic (EMG) responses and resulting limb movement were investigated. In ketamine-anesthetized cats, paw movement kinematics in 3D and EMG activity from 8 to 12 forelimb muscles evoked by ICMS applied to the forelimb area of the cat motor cortex (MCx) were recorded. The EMG responses evoked by ICMS were also compared to those evoked by focal ictal bursts induced by the iontophoretic ejection of the GABA_A_ receptor antagonist bicuculline methochloride (BIC) at the same cortical point. The effects of different initial limb starting positions on movement trajectories resulting from long-duration ICMS were also studied. The ICMS duration did not affect the evoked muscle activation pattern (MAP). Short (50 ms) and long (500 ms) stimulus trains activated the same muscles in the same proportions. MAPs could, however, be modified by gradually increasing the stimulus intensity. MAPs evoked by focal ictal bursts were also highly correlated with those obtained by ICMS at the same cortical point. Varying the initial position of the forelimb did not change the MAPs evoked from a cortical point. Consequently, the evoked movements reached nearly the same final end point and posture, with variability. However, the movement trajectories were quite different depending on the initial limb configuration and starting position of the paw. The evoked movement trajectory was most natural when the forelimb lay pendant ~ perpendicular to the ground (i.e., in equilibrium with the gravitational force). From other starting positions, the movements did not appear natural. These observations demonstrate that while the output of the cortical point evokes a seemingly coordinated limb movement from a rest position, it does not specify a particular movement direction or a controlled trajectory from other initial positions.

## Introduction

The discovery that ICMS trains having a duration of the order of typical movements (Graziano et al., 2002a) was a remarkable finding that led to the idea that stimulation of a single point in the MCx evokes coordinated movements and behaviorally meaningful actions (Graziano et al., 2002b; Graziano and Cooke, 2006). Similarly, in the cat and rat, coordinated forelimb movements can be evoked by ICMS of the MCx (Ethier et al., 2006; Bonazzi et al., 2013). In the cat, these movements have a bell-shaped velocity profile, and the movement amplitude is a sigmoid function of stimulus intensity (Ethier et al., 2006). It has also been reported that long-train ICMS of the posterior parietal cortex (PPC) of prosimian galagos evokes ethologically significant behaviors such as hand-to-mouth and defensive movements (Stepniewska et al., 2005). In the experiments of Ethier et al. (2006), the animals' forelimb laid pendant ~ perpendicular to the ground before ICMS was applied, whereas in those of Bonazzi et al. (2013), the forelimb was at an angle of approximately 45 degrees down from the horizontal position. In the galago study, neither the initial position of the forelimb was reported nor the movement trajectories were measured. Any sustained muscular activation evoked by ICMS will bring the forelimb to a spatial location where it will be in equilibrium with the gravitational force. At this final location, the limb will have a given posture, or geometric configuration, dependent on the relative tonic activities of the muscles activated by the ICMS. It is therefore not clear whether a single locus of stimulation, in the MCx or PPC, is capable of evoking a truly coordinated movement along a controlled trajectory, as in natural movements. These considerations also apply to the study of Graziano et al. (2002a) on macaques, in which the initial position of the hand in space was due to voluntary activity prior to ICMS. Furthermore, the use of long-duration intracortical stimulus trains has been criticized. For example, Strick (2002) suggested that high currents and long-duration stimulus trains may activate an area well-beyond the stimulation locus, a point that has yet to be tested.

To these ends, the muscle activation patterns (MAPs) evoked by conventional ICMS were compared to those evoked by long-duration ICMS. Representing MAPs as vectors of integrated EMG (IEMG) values allows for a quantitative comparison of their similarity, as will be explained. In addition, MAPs evoked by ictal bursts of neural activity were compared to those evoked by ICMS at the same cortical point. Ictal bursts occur when local GABAergic synaptic transmission is reduced by iontophoretic ejection of GABA_a_ receptor antagonists, such as bicuculline methochloride (BIC) (Schneider et al., 2002; Capaday and Rasmusson, 2003; Ethier et al., 2006). This produces nearly periodic bursts of neural activity. When these bursts are of sufficient amplitude and duration, the resulting corticospinal drive evokes muscle activation and limb movement. Last, the characteristics of limb movements evoked by long-duration ICMS such as movement direction, limb trajectory, and final position attained were also determined.

## Materials and methods

The data reported herein were obtained from experiments on eight male cats weighting between 3.3 and 4.9 kg. The study was approved by the local ethics committee of Laval University, where the experiments were conducted at the time the author was on the faculty. The experiments conformed with the procedures outlined in the Guide for the Care and Use of Laboratory Animals, published by the Canadian Council on Animal Protection.

### Animal preparation

Details on surgical procedures, electrophysiological methods, and homeostatic measures used in the present study can be found in previous reports from this laboratory (Capaday et al., 1998; Schneider et al., 2001, 2002; Capaday and Rasmusson, 2003; Ethier et al., 2006). In brief, the animals were anesthetized with an intramuscular injection of ketamine (33 mg/kg) and xylazine (1 mg/kg). Once the surgical procedures were terminated, a perfusion pump was connected to a cannula in the femoral vein, and a steady flow of ketamine (10–30 mg/hr, depending on the animal) was delivered throughout the experiment. The animals' temperature was maintained near 37^0^ C by a heating blanket wrapped around the animals' trunk and by an overhead heat lamp, when needed. The blood pressure was maintained at about 100 mmHg. A long skin incision was made to expose the muscles of the left forelimb and shoulder. A pair of multi-stranded, stainless steel wires, separated by ~1.5 cm, was inserted in eight to 12 of the following muscles, depending on the animal: the flexor digitorum profundus (FDP), flexor carpi radialis (FCR), palmaris longus (PL), extensor carpi radialis longus and brevis (ECRl and ECRb), lateral head of the triceps (TriL), brachialis (Br), clavobrachialis (ClBr), brachioradialis (Brad), biceps (Bi), teres major (TM), latissimus dorsi (LD), spinodeltoid (SpD), pectoralis major (PMj), and pectoralis minor (PMn). The EMG signals were amplified by a factor of 1,000, high-pass-filtered at 20 Hz, rectified, and low-pass-filtered at 1,000 Hz.

### Microstimulation procedures

Stainless steel microelectrodes ranging in impedance from 800 kΩ to 1.2 MΩ were used to microstimulate the forelimb area of MCx. Trains of stimuli, ranging from 50 to 800 ms in duration, were delivered at random intervals between 2 and 7 s in layer V of the right MCx. The duration of single pulses was 0.2 ms, and the stimulus rate was 333 Hz. The output of each cortical point in terms of evoked EMG activity and limb movement (see below) was determined as a function of stimulus intensity and train duration. The input–output characteristics of each cortical point were characterized by first determining the threshold and then by varying the stimulus intensity from threshold to a maximum of 70 μA, in random increments. The effects of the stimulus train duration on evoked EMG activity and movement were also determined by varying it from 50 to 500 ms and, in some cases, up to 800 ms, in a random order.

### Disinhibition of cortical points

BIC was dissolved at a concentration of 10 mM in distilled water and ejected from micropipettes having tip diameters of about 2–3 μm with a positive current of 40–60 nA. The resulting ictal bursts ceased after about 1 h.

### Analysis of evoked EMG activity

The microstimulation-evoked EMG activity was sampled at 2 KHz. The duration of the sampled sweeps was 1,000 ms, including a 100-ms pre-stimulus period. Typically, eight sweeps were sampled for each stimulus intensity and duration combination, averaged in real time and stored on hard disk. The integral of the rectified evoked EMG (IEMG) activity was calculated for each muscle. Any background EMG activity preceding the stimulus was subtracted from the responses. These integrated EMG values were expressed as eight, or 12, dimensional vectors, depending on the number of EMG recordings. The EMG response vector will be referred to as the muscle activation pattern (MAP) (Valero-Cuevas, 2000; Ethier et al., 2006). Each element of a MAP vector is the net evoked IEMG activity of a given muscle. For a cortical point *p*, the MAP vector $\vec{m_{p}}$ is defined as follows:


(1) $$\begin{array}{l} \vec{m_{p}}=IEMG_{1}\cdot \vec{m_{1}}+IEMG_{2}\cdot \vec{m_{2}}+\ldots +IEMG_{n}\cdot \vec{m_{n}} \end{array}$$

where *IEMG*_*i*_ is the value of the integrated EMG activity of the *i*^*th*^ muscle and $\vec{m_{1}},\vec{m_{2}},\ldots ,\vec{m_{n}}$ are orthogonal unit vectors that span the muscle space. To determine the similarity (i.e., correlation) between any two MAPs, the vectors were transformed to zero mean by subtracting the mean value of the raw vector. The correlation between any two vectors $\vec{m_{a}}$ and $\vec{m_{b}}$, or equivalently, the difference in their direction θ was determined from their dot product. For zero-mean vectors, the dot product is identical to the Pearson product–moment correlation coefficient *r* given as follows:


(2) $$\begin{array}{l} r=cos\left(\theta \right)=\frac{\vec{m_{a}}\cdot \vec{m_{b}}}{\left(\left|\vec{m_{a}}||\vec{m_{b}}\right|\right)} \end{array}$$

where $\left|\vec{m_{a}}\right|$ and $\left|\vec{m_{a}}\right|$ are the Euclidean vector magnitudes. The IEMG value of each muscle in a given raw MAP vector $\vec{m_{p}}$ was plotted against the stimulus intensity, and a Boltzmann sigmoid function was fitted as described in Devanne et al. (1997).

### Paw kinematic measurements and analysis

The kinematics of the microstimulation-evoked movements were measured by a small (1 x 2 cm) 6D electromagnetic sensor (Polhemus, Inc.) placed on the tip of the paw (Ethier et al., 2006). The kinematic signals were digitized at 120 Hz. Only the position coordinates in three orthogonal (x, y, and z) directions were used in the present analysis. The body of the cat was laid on a cushion with its forelimbs hanging perpendicular to the ground and free to move in all directions against gravity. Data acquisition was triggered at the onset of stimulation and included a 100-ms pre-stimulus interval. The trials starting from the pendant position of the forelimb will be referred to as the control trials. The final position attained by the paw was determined at the time the tangential velocity reached 0. The coordinates of the final position attained by the paw were calculated by averaging the results obtained in four to eight trials. The variability, measured as a standard deviation, was also calculated along each coordinate axis. Test trials involved moving the initial position of the paw. The experimenter moved and maintained the paw at random positions within the limb's natural workspace. The paw rested on the tip of the index finger of the experimenter and moved away naturally when the forelimb musculature was activated by ICMS. Care was taken not to hinder the stimulus-evoked paw movement. In the control trials, the limb was unsupported. The final position attained in the test trials and its variance were calculated as for control trials. The mean Euclidean distance and variability of paw positions between the test and control trials were calculated. Further details of the movement kinematic analyses are described in the results.

## Results

The results are presented in four sections. First, the effects of ICMS intensity on evoked MAPs and limb movements are described. Second, it will be shown that at equal stimulus intensity, the MAP evoked from a motor cortical point by short-duration (50 ms) ICMS trains is highly correlated with that evoked by long-duration (500 ms) ICMS trains. Third, MAPs evoked by focal ictal bursts are highly correlated with those evoked by ICMS of the same cortical point. Finally, the effects of varying the initial position of the paw and consequently the forelimb configuration on MAPs and movement trajectories evoked by ICMS are described.

### MAPs depend on stimulus intensity

**Figure 1:**
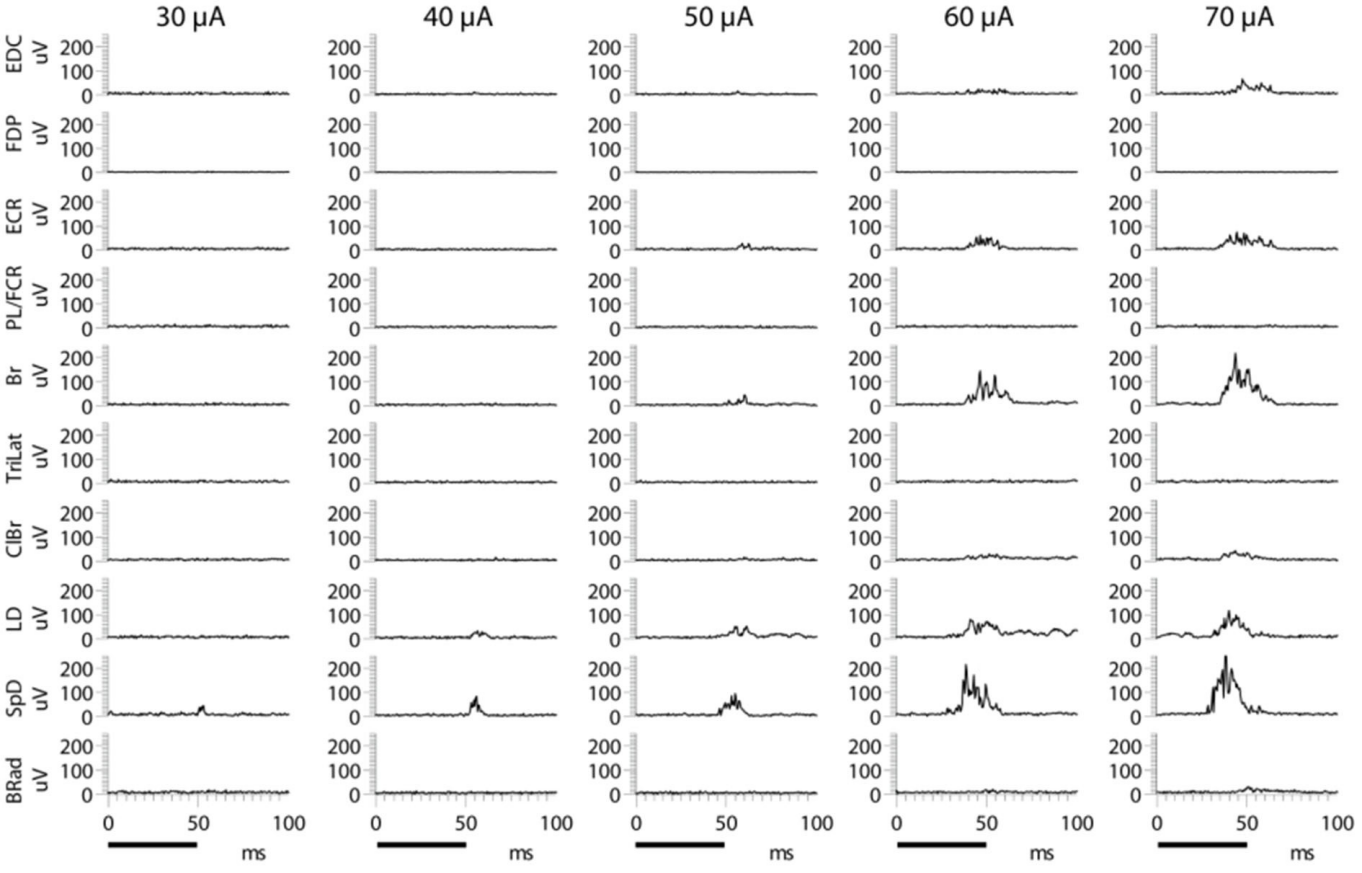
Examples of evoked EMG activity following microstimulation of a motor cortical point at different current intensities. The train duration was 50 ms, as indicated by the black bars at the bottom of the figure. Increasing stimulus intensity produces larger responses in muscles recruited at lower intensity, as well as a gradual recruitment of more muscles. Note the simultaneous appearance of Br and ECR at 50 μA.

**Figure 2:**
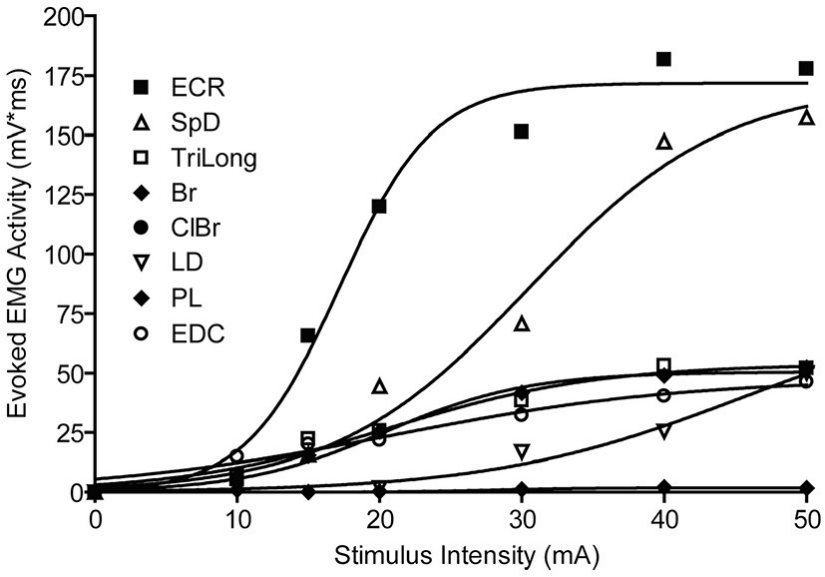
Recruitment curve of muscle activity vs. stimulus intensity. The evoked EMG responses are fitted by a Boltzmann function, bottom constrained to 0, r^2^ range = [0.97–0.98]. Different muscles have a different threshold, rate of rise, and plateau values.

A typical example of the effects of stimulus intensity (duration 50 ms) on the evoked MAP is shown in Figure 1. In this example, only one muscle was activated near threshold (SpD, ~30 μA). In all cases, 67 cortical points in eight cats, the evoked EMG activity ceased at nearly the time of stimulus termination. Increasing the stimulus intensity from threshold to 70 μA resulted in a larger SpD response, as well as the progressive recruitment of more muscles. In ~40% of cases, it was not possible to obtain a response in only one muscle at the stimulus threshold. For example, activity in the ECR and Br was often evoked at the threshold intensity. Similarly, it was also observed that as stimulus intensity increased, the threshold at which new muscles were recruited was not always distinct for each additional muscles recruited, that is, more than one muscle was often recruited at the same threshold. An example of this can be seen in Figure 1, where the Br and ECR muscles were simultaneously recruited at 50 μA. This can also be seen in Figure 2, where an example of the relation between IEMG activity and stimulus intensity is shown. Note that several muscles were recruited at the stimulus threshold (10 μA) and that as the stimulus intensity was increased, more than one muscle was sometimes recruited at the same stimulus intensity (e.g., Bi, SpD, and TM at 22 μA). The relation between muscular activity and stimulus intensity was approximately sigmoidal for all 67 cortical points studied in eight animals, on average ~ 8 cortical points per animal. The R^2^ values of the Boltzmann function data fits were typically 0.90, with a range of 0.88–0.98.

### MAPs are dependent on stimulus intensity, but not on duration

**Figure 3:**
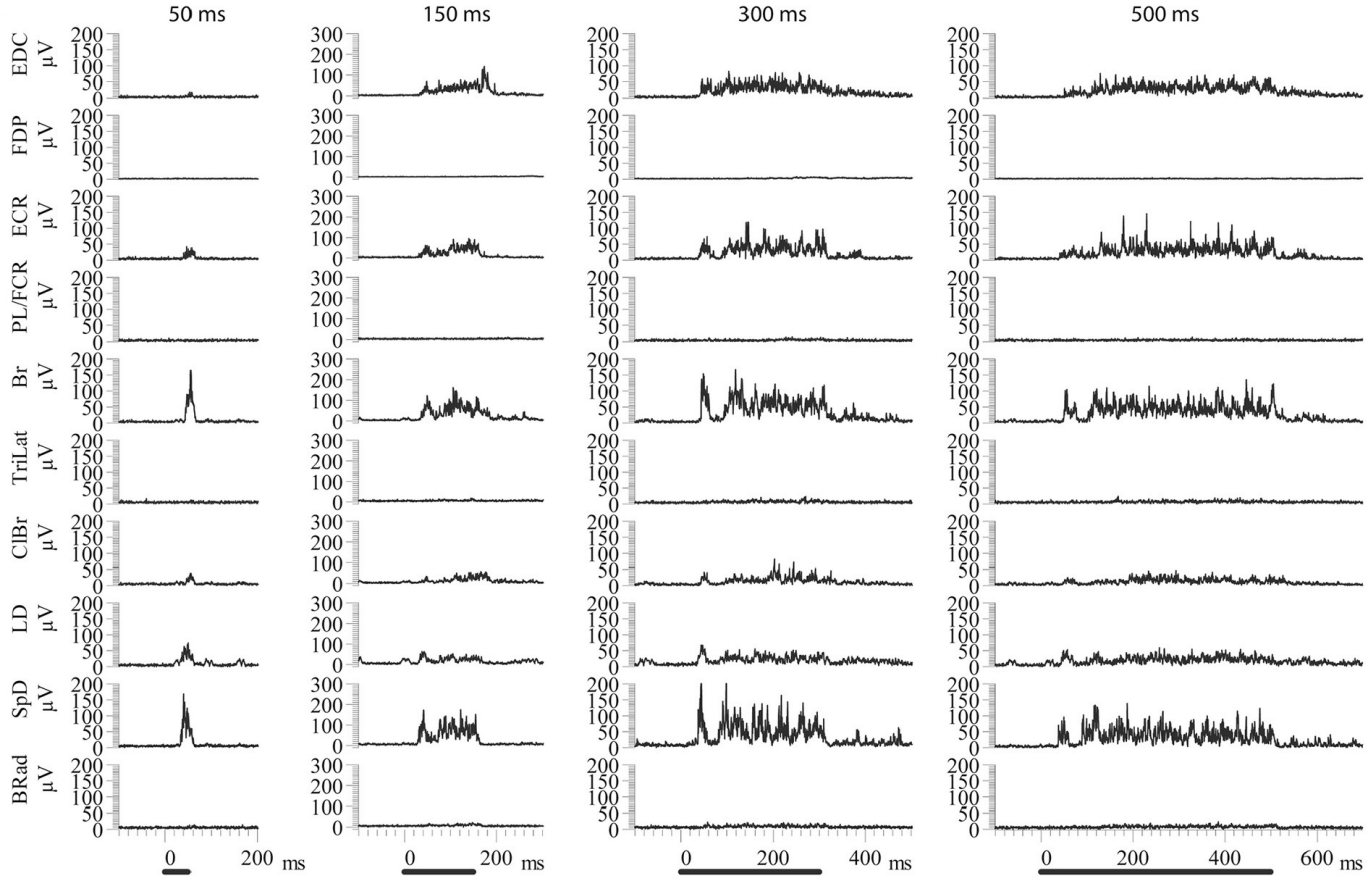
Examples of ICMS evoked EMG activity using constant current amplitude (50 μA) and different train durations. The same muscles are activated in each condition in approximately the same relative proportions. In all cases, the evoked EMG activity ceases near the time of stimulus termination. Back bars indicate the onset and duration of stimulation.

**Figure 4:**
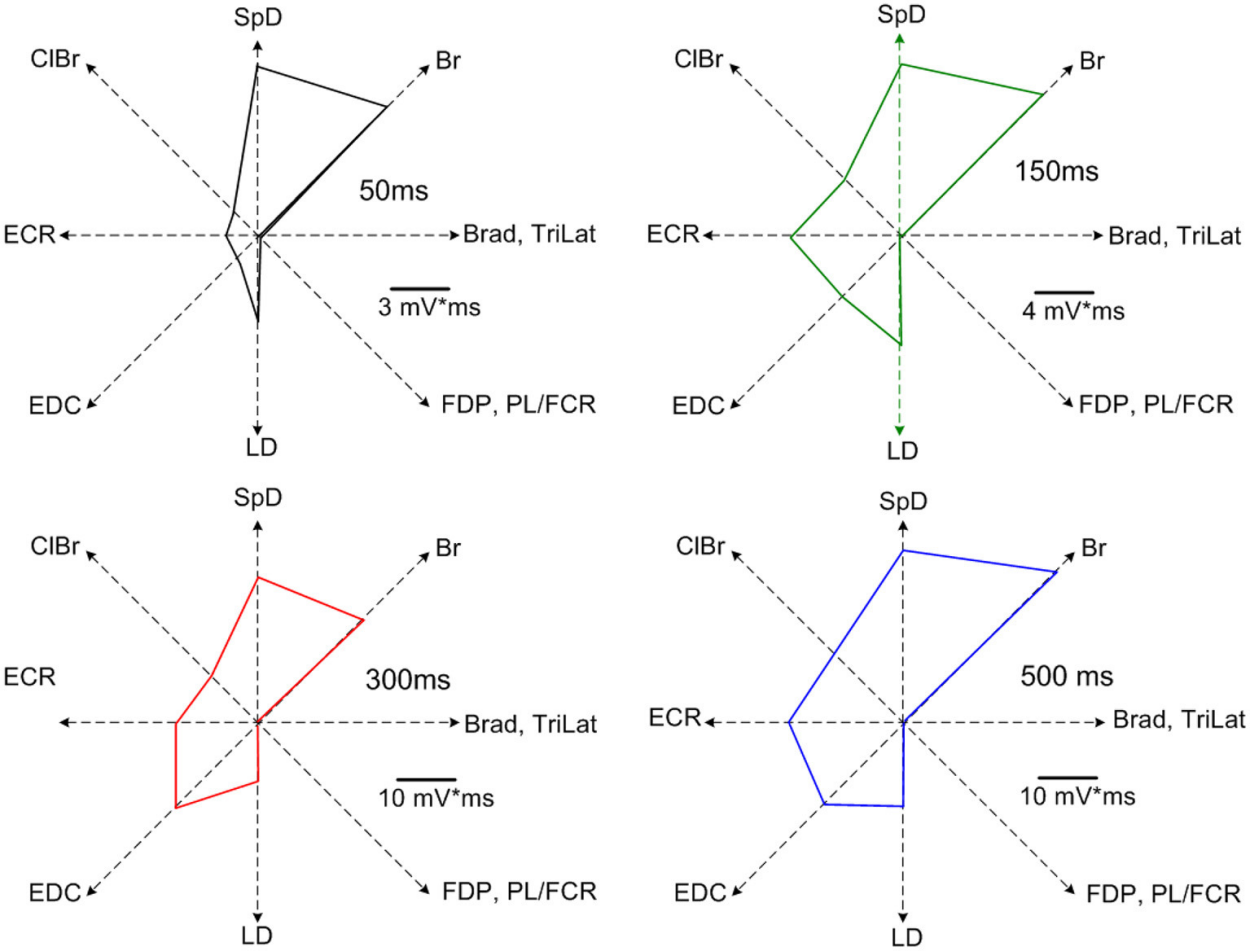
MAPs corresponding to the data of Figure 3. Note the highly similar recruitment pattern and the absence of recruitment of muscles antagonistic to the recruited muscles at all stimulus durations (i.e., TriLat, FDP, and PL/FCR). The average correlation coefficient of the MAP correlation matrix was r = 0.91 (SD= 0.12, *p* = <0.001).

Increasing the stimulus duration increased the duration of EMG activation. Importantly, the recruited muscles were the same for all stimulus durations, as can be seen in the example shown in Figure 3. Note once again that the EMG responses of the various muscles in the MAP ceased at nearly the time of stimulus termination. Neither the MAP nor the relative magnitudes of activity in each muscle changed by varying the stimulus duration (Figure 3). Only the response duration is affected by a change in the stimulus duration, but not the MAP (Figure 4). Thus, the same muscles in roughly the same relative proportions are activated, independently of the stimulus duration. Importantly, the muscles antagonistic to the recruited ones, such as FDP and TriLat, remained inactivated for all stimulus durations. The MAPs corresponding to each stimulus duration are shown in Figure 4. The average correlation coefficient of the MAP correlation matrix was r = 0.91 (SD = 0.12, *p* = <0.001). Considering 23 such observations (~ four per animal) obtained in five animals, comparing 50-ms vs. 500-ms stimulus trains, the average correlation coefficient between the evoked MAPs was r = 0.88 (SD= 0.17, *p* < 0.01). In summary, in contrast to increasing stimulus intensity, increasing stimulus durations did not lead to the recruitment of new muscles, including their antagonists.

### Ictal cortical bursts evoke essentially the same MAP as ICMS

**Figure 5:**
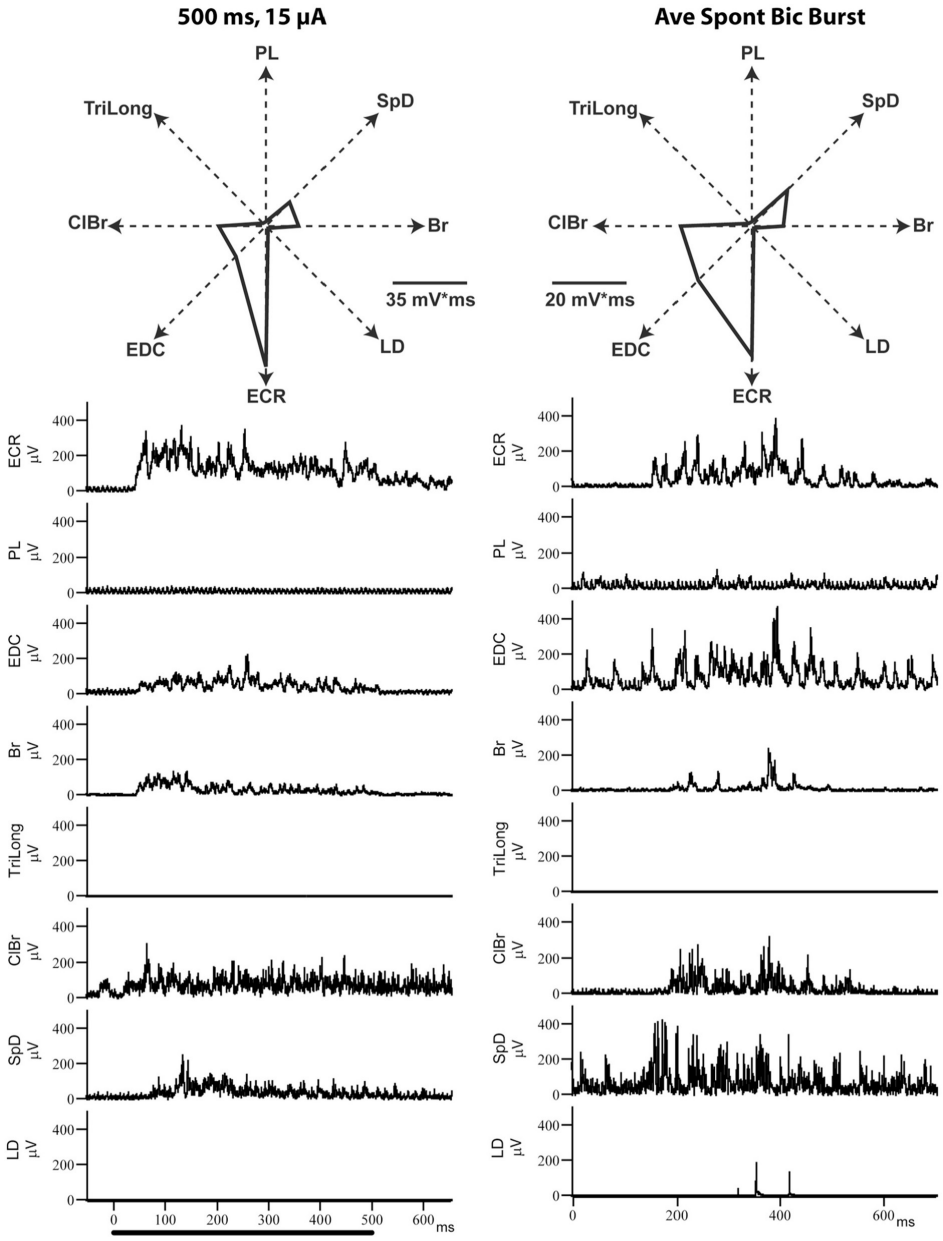
Example of ICMS (left)- and bicuculline (right)-evoked EMG activity from the same motor cortical point. Each of the EMG traces represents the average of 8–12 individual responses. Note how similar the evoked MAPs are, despite the very different mechanisms of neural activation.

In this series of experiments, the MAP evoked by ICMS at a given cortical point was first determined and compared to that evoked by focal ictal bursts produced by BIC iontophoretically ejected at the same point. As show in Figure 5, the MAPs evoked by BIC and ICMS are very similar, as can be seen from the EMG recordings, or their polar plot representations. Considering the four cortical points explored in two cats, the average correlation coefficient of the MAPs evoked by each activation method was r = 0.90 (SD= 0.1, *p* < 0.01).

### Final paw position is relatively independent of the initial paw position, but not movement direction and trajectory

**Figure 6:**
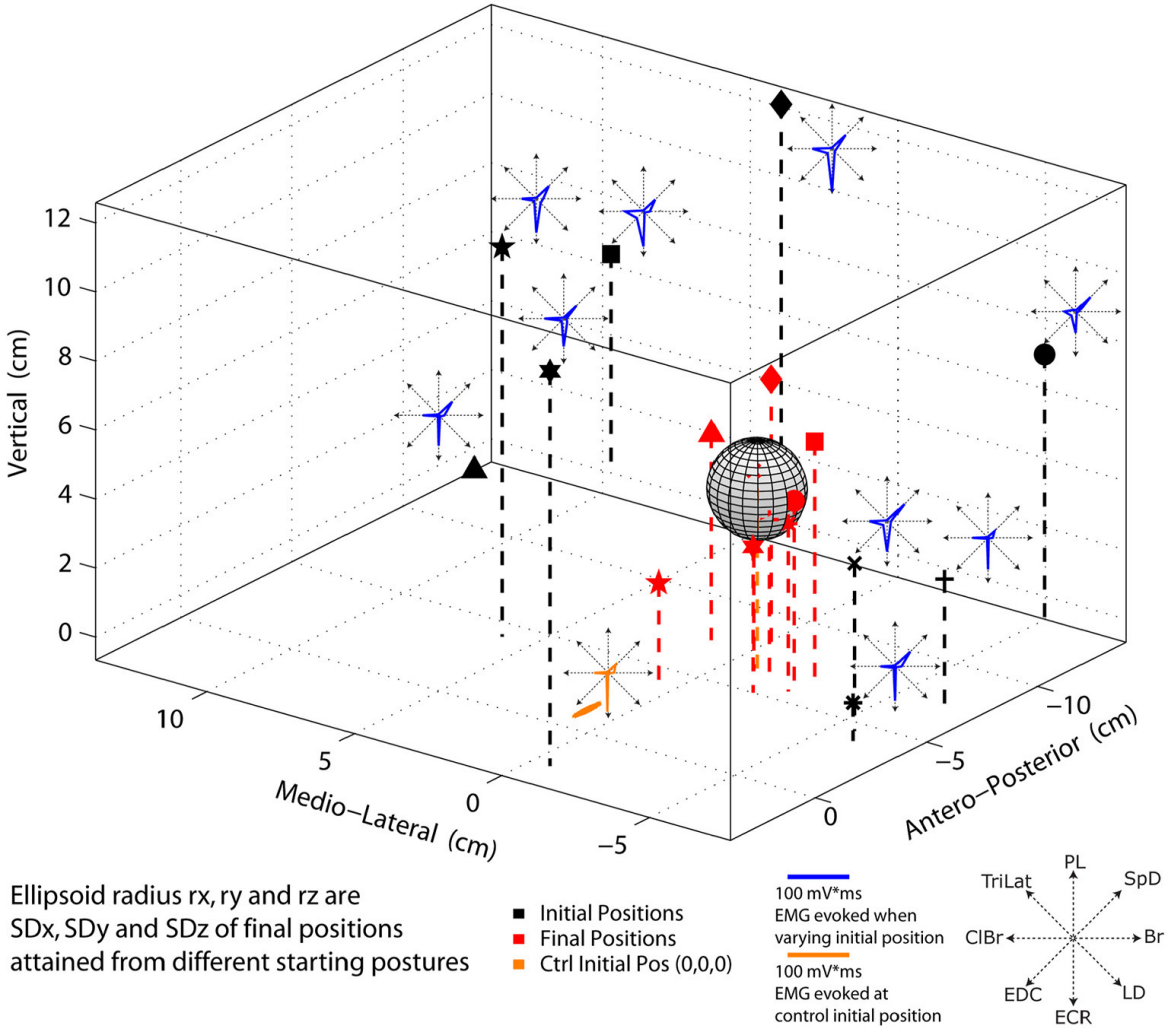
Polar plots of EMG activity and 3D representation of initial and end point position evoked by 60 μA, 800 ms ICMS of the same cortical point. Black markers (stars, diamond, triangle, etc.) represent the initial position where the paw was placed before stimulation. Red markers indicate the corresponding final position attained by the movement. MAPs evoked from different initial positions are shown as insets in blue. The control MAP with the forelimb hanging approximately straight and vertical to the ground is shown in orange The ellipsoid of standard deviations for the test movements (i.e., from different initial positions of the paw) is shown in gray. The red dots within the ellipsoid are the final positions attained from the control condition.

**Figure 7:**
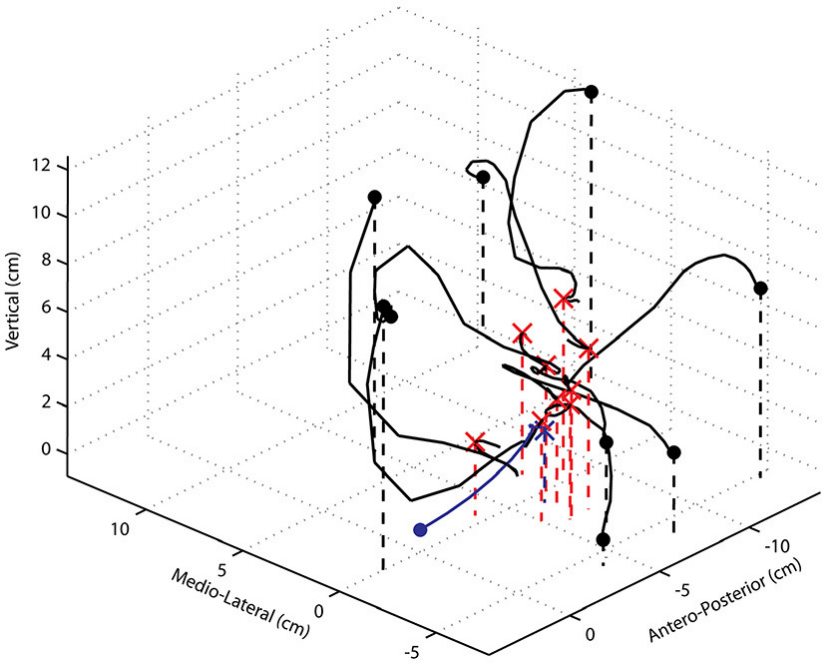
Paw trajectories of movements evoked by 60 μA, 800 ms ICMS trains starting from widely different initial positions. The thick black dots represent different initial paw positions, and the red x markers indicate the final position attained in each case. The movement trajectory starting with the forelimb freely hanging ~ perpendicular to the ground is shown in blue. Note the changes and reversal of directions in many of the test examples and the strong vertical component of the movement trajectory when the paw is placed above the mean final position attained in control trials.

The direction and trajectory of the paw movement evoked by long-duration ICMS of a cortical point depended on the initial limb position, but the final positions attained were statistically indistinguishable. The details of the statistical results are given further as follows. In the example of Figure 6, nine widely different initial positions of the paw and the corresponding final positions attained are shown. The ICMS parameters in all cases were 60 μA in intensity and 800 ms in duration. Note that in this example and that in Figure 7, the data shown are single trials, and not averages. The mean final position attained in nine trials is shown as a gray ellipsoid. The ellipsoid is centered at the average final position attained, and its radii along the x-(medio-lateral), y (antero-posterior), and z (vertical) axes correspond to the standard deviation in each respective direction. The average final position attained during the control trials (i.e., with the forelimb hanging pendant ~ vertical to the ground) is within the ellipsoid, and small red dots indicate the final position attained for each trial (Figure 6). The mean Euclidean distance of all test trials with varied initial positions and control trials (from the same initial position) was 1.0 cm (sd = 2.1 cm, *n* = 45). The mean distances for the varied initial position trials from the mean end point of the control trials along the x, y, and z axes were, respectively, 3.5, 7.1, and 6.8 mm. In the same order, the mean variability of distances relative to the initial position of control trials was 1.3, 1.12, and 1.13 cm, respectively. Welch's *t*-test based on 45 observations in two animals (~22 per animal) revealed no statistical differences in the final position attained along any of the coordinate axes (*p* > 0.4). Similarly, despite wide differences in the starting position of the paw, the evoked MAPs, dominated in this example by ECR, ClBr, and SpD, were remarkably alike, as shown by the inset polar plots of Figure 6. The similarity between the MAPs was quantified. The average correlation coefficient between the test MAPs and the respective control MAPs, 45 observations in two animals, was r = 0.92, SD = 0.08. This demonstrates that the MAP evoked from a cortical point has little, if any, dependence on the initial position of the paw.

An example of the paw trajectories starting from 10 widely different initial positions of the paw are shown in Figure 7. In all cases, the paw moved away from the experimenter's supporting finger driven by the ICMS-evoked muscle activities. There are several noteworthy points concerning the characteristics of these trajectories. First, the initial direction was not always in the direction of the final end point. Second, for the initial conditions where the paw started above the final position attained in control trials, the forelimb moved passively downward, and thereafter, the evoked muscular forces overcame the gravitational force and brought the paw to its final position. Thus, only the initial and latter parts of the movement trajectory were driven by evoked muscle activity. Third, the trajectories are not straight, or even close to straight, and there are several changes in direction as well as reversals of direction, these being most obvious for movements starting either more medially, or laterally, to the mean control final position. Here too, only the initial and latter parts of the movement were driven by evoked muscle activity. Thus, while from the control position with the forelimb pendant and approximately vertical to the ground, the evoked movements appear coordinated, fairly straight, and seemingly purposeful, and from other starting positions, the movements were clearly unnatural.

## Discussion

Three principal sets of observations on the nature of MCx outputs were presented. First, a cortical point contains the representation of several synergistic muscles that are revealed by increasing stimulus intensity. Second, the MAP evoked by ICMS of a single cortical point is independent of stimulus duration. Third, a single cortical point can evoke a seemingly coordinated movement of the forelimb when it is pendant and thus initially in an equilibrium configuration with respect to the gravitational force. However, from out of equilibrium starting positions, the forelimb does not follow controlled trajectories but reaches a similar end position as from the pendant position. The term controlled trajectory is used to mean that in going from one position to another, limb motion is entirely driven by coordinated muscle activity, which includes components to accelerate, decelerate, and hold the limb in place. Thus, from the out of equilibrium starting positions, the free fall downward motion of the forelimb changes in direction, and direction reversals observed before muscle forces develop sufficiently to overcome gravity are not controlled movements. The interpretations and implications of these observations are discussed in turn.

Despite a 10-fold increase in stimulus duration, the evoked MAPs remain correlated, that is, the same muscles are recruited in nearly the same relative proportions. This demonstrates that increasing stimulus duration does not cause greater stimulus spread, as was suggested by Strick (2002). If this were the case, the MAPs would be expected to change, but they do not. By contrast, increasing stimulus intensity does recruit additional muscles; MAPs thus depend on stimulus intensity. Importantly, the evoked MAPs represent coordinated synergistic muscle activation patterns, as evidenced by the fact that at least from the pendant position of the forelimb, a coordinated and seemingly purposeful movement is evoked by long-duration ICMS trains. Furthermore, reciprocal inhibition between antagonistic muscles is a basic rule of ICMS-evoked MAPs (Ethier et al., 2007). For example, triceps is never recruited during Br activity, and the PL/FCR is never recruited during ECR activity (Figures 1, 3). However, considering shoulder muscles, ICMS can evoke co-contractions of antagonistic muscles in both cat (Ethier et al., 2007) and monkey (Griffin et al., 2011). This may reflect the fact that the shoulder acts as a base of support for the forelimb.

The MAPs evoked by ICMS, or ictal bursts, at the same cortical point are highly correlated, the same muscles are recruited in nearly the same relative proportions. This result thus shows that the two methods reveal the representation of muscles within the activated cortical locus. This is discussed in greater detail in further text. However, the asymmetry of cortically mediated spinal reciprocal inhibition significantly biases ICMS-evoked MAPs in favor of physiological flexors (Ethier et al., 2007). In that study, following an intravenous injection of a single bolus of strychnine, ICMS of a cortical point at which only a physiological flexor was previously activated also elicited simultaneous activation of its antagonist. This demonstrates that even at a single cortical locus, antagonistic corticospinal neurons can be closely grouped, or intermingled, as are corticospinal neurons that control forelimb muscles acting at different joints (Schieber and Hibbard, 1993; Schneider et al., 2001; Rathelot and Strick, 2006; Capaday et al., 2009). Taken together, these observations explain why evidence for a columnar organization of MCx has been weak (Kalaska and Rizzolatti, 2013). The intermingling of corticospinal neurons that control different muscles is inconsistent with a columnar organization of individual muscles. Thus, contrary to the well-established columnar organization of primary sensory areas, this is not a feature of motor cortex architecture. In addition to the intermingled representation of muscles within a cortical locus, muscles are represented many times over across the MCx, in non-contiguous loci and in various combinations with other muscles (Donoghue et al., 1992; Schneider et al., 2001; Devanne et al., 2006; Rathelot and Strick, 2006; Capaday et al., 2009).

The axon collaterals of cat MCx neurons extend up to 6–7 mm away from their soma and are studded with synaptic boutons all along their course (Capaday et al., 2009), forming a recurrent network connectivity. The dense core of synaptic bouton connectivity identified by Capaday et al. (2009) in the forelimb area of the cat motor cortex covers an area of ~2.0 mm^2^ (*radius*~0.8 *mm*) and contains the representation of muscles that act on different joints. Based on the chart developed by Ranck (1975), at a maximal stimulus intensity of 70 μA used in this study, ICMS current spreads over a radius of about 500 μm from the tip of the microelectrode, consistent with independent calculations (Tehovnik et al., 2006; see also Histed et al., 2009; Van Acker et al., 2013). Autoradiographic and electrophysiological experiments have shown that iontophoretic ejection of bicuculline diffuses over a radius of about 500–600 μm from the tip of the micropipette (Jacobs and Donoghue, 1991; Schneider et al., 2002; Ethier et al., 2006; Capaday et al., 2011). The resultant ictal neural bursts activate spiking in a surrounding area of ~1.5 mm radius (Capaday et al., 2011). It is clear from these combined anatomical and physiological measurements that no two points within the MCx, at least 1.5 mm in radial distance and up to 6–7 mm, are functionally independent. More importantly, the microstimulation current spread is limited to the area of strongest synaptic connectivity between neurons, the dense core. ICMS thus recruits corticospinal neurons, either directly or synaptically, within the dense core of connectivity. Antidromic activation of neurons outside the dense core may be possible, given the recurrent synaptic connectivity of points up 6–7 mm apart. However, as stated earlier, ictal bursts of maximal intensity activate orthodromically an area of ~1.5 mm radius, roughly twice that of the dense core. Yet, the resultant MAP is highly correlated with that evoked by ICMS of the same point. The observation that two very different methods of activation of the cortical circuitry give essentially the same result is remarkable and demonstrates that ICMS recruits intermingled corticospinal neurons within the dense core of connectivity.

Do the observations of Graziano et al. (2002a) and the many subsequent studies of others (e.g., Stepniewska et al., 2005; Ethier et al., 2006; Bonazzi et al., 2013) demonstrate that the output of a single cortical locus encodes a particular movement? The hypothesis developed by Graziano's group states that “… *activity at a site in motor cortex acts as a higher-order signal, instructing the limb to move to a certain posture regardless of the initial posture*” (Graziano et al., 2002a,b, 2004). The experimental results presented here are consistent with this hypothesis, but do not necessarily support it. More importantly, whether the evoked movement from initial to final positions follows a controlled trajectory is addressed neither by the hypothesis nor by the observations upon which it is based. Here, it was shown that in all cases where the initial paw position was away from the control pendant position, part of the movement was due to the gravitational force. The paw, therefore, did not follow a controlled spatial trajectory. Thus, while a single cortical point can drive the paw to a given spatial location relative to the animal's body and maintain the forelimb in a given posture, it does not generate the neural drive necessary for a controlled movement trajectory. One may ask whether activation of at least two cortical points, as was performed in the study of Ethier et al. (2006), is sufficient to generate a controlled trajectory. In that study, it was shown that the simultaneous activation of two motor cortical points evoked a movement that was a blend of those evoked by each cortical point activated on its own and that the resultant movement direction followed the rules of linear algebra. However, because the forelimb was initially in the pendant position in all cases tested, the results of that study cannot be used to answer the question; therefore, further experiments are required. It should be noted that the results shown in Figure 7 of the present study are similar to those in Figure 2 of Graziano et al. (2002b). Despite the single frontal plane view presented in that figure and a low video frame rate of 30 Hz, it can be observed that the hand paths are curved with changes and reversals of direction and the passive influence of the gravitational force on the movements can be inferred.

The variability of final positions attained, whether from the control pendant position, or from the out of equilibrium starting positions, can be explained by the fact that the magnitude of MAPs varies from trial to trial (see the EMG polar plot insets in Figure 6). The fact that MAPs are highly correlated does not imply that their magnitude is the same, explaining the trial-to-trial variability of the final position attained. It should be noted that there are no proprioceptive inputs to the cat MCx under ketamine (e.g., see Capaday and Rasmusson, 2003). Furthermore, ICMS would “hijack” proprioceptive inputs (Griffin et al., 2011), meaning that neural activity at the point of stimulation is only driven by the current pulses. It is therefore not surprising that MAPs were not dependent on the forelimb position and posture. MAPs thus depend on the local neuronal representation of muscles at a cortical point and their corticospinal connections including those on spinal interneurons. Under ketamine anesthesia, phasic stretch reflexes can be elicited by rapid flexion/extension of a joint, but not tonic stretch reflex activity. Therefore, there was no tonic activity in the forelimb muscles at the time of ICMS. Spinal interneurons are controlled by the MCx, leading to, for example, reciprocal inhibition with a stronger bias from physiological flexors to extensors, as explained previously. Thus, any spinal interneuronal bias resulting from afferent inputs as a result of a change in limb configuration will be modulated by the descending activity evoked by ICMS. Differences in the magnitude of MAPs may be due in part to proprioceptive inputs at the spinal level (e.g., Mussa-Ivaldi et al., 1990; Lemay and Grill, 2004), but these do not change MAP components (i.e., recruited muscles). Indeed, commenting on their intraspinal ICMS experiments, Mussa-Ivaldi et al. (1990) state “… changing the leg's configuration only modestly affected the EMG response to the stimulation” (e.g., see their Figure 4). The data presented here are consistent with the intraspinal ICMS experiments and clearly show that the MAPs elicited by activation of a cortical point are highly correlated, despite changes of initial limb configuration.

We found no instances that changing the posture of the limb caused the EMG output to switch from flexor to extensor muscle activity, or vice versa. The apparent reversals from flexor to extensor muscle activity, or vice versa, reported by Graziano et al. (2002a, 2004) can be most parsimoniously explained by the actions of spinal neural mechanisms, rather than by special properties of the MCx. Stretch of the triceps, for example, would *via* the stretch reflex pathway inhibit biceps α-motoneurons. Consequently, the ICMS-evoked EMG activity in biceps would be reduced, and vice versa. Furthermore, in conditions that allow for tonic stretch reflex activity, sufficient stretch of the triceps can result in an evoked triceps EMG response, and vice versa. Such reversals are commonly seen in transcranial magnetic stimulation experiments in humans (e.g., see Capaday, 1997). In both cases, the underlying reason is that the evoked descending volley is composed of spikes in corticospinal axons innervating different motoneuron pools.

## Conclusion

Neural activation produced by long-duration ICMS does not spread beyond that of conventional ICMS. MAPs evoked by ICMS or focal ictal bursts of neural activity at the same cortical point are strongly correlated, despite differences in the mechanisms of neural activation. Taken together, these observations demonstrate that ICMS reveals which muscles are represented at a given cortical point. However, the asymmetry of the strength of cortically mediated reciprocal inhibition between antagonistic muscles strongly biases the content of MAPs toward physiological flexors. This also biases the type of movements that can be evoked by long-duration ICMS. In the cat, for example, extension movements of the forelimb away from the body are rarely evoked by long-duration ICMS. MAPs do not change with initial paw position, or equivalently with the initial limb geometry. Consequently, movements evoked by long-duration ICMS reach nearly the same final position, with variability, despite widely different starting positions. This observation neither supports or contradicts existing theories of motor control. It is a simple fact that the forelimb will adopt a given posture determined by the balance of muscular and gravitational forces. However, the movement trajectories differ considerably from one initial position to another. This demonstrates that in natural conditions, movement trajectory depends on the coordinated activation of a multitude of cortical points, terminating at a final locus of motor cortical activity, which holds the limb at a spatial location. This idea is consistent with several lines of evidence showing that wide areas of the MCx are activated even during simple coordinated movements (e.g., see Georgopoulos and Kalaska, 1982; Amassian et al., 1995; Sanes and Schieber, 2001; Devanne et al., 2002). The activation sequence is likely to be based on proprioceptive information conveyed directly to the MCx, or from higher cortical areas. Thus, while the results of Graziano et al. (2002a; 2002b; Aflalo and Graziano, 2007) may be interpreted as a map of postures contained in the MCx, it is not a map of movements. The mechanisms of timing and selection of motor cortical loci to produce coordinated movements remain to be elucidated.

## Data availability statement

The raw data supporting the conclusions of this article will be made available by the authors, without undue reservation.

## Ethics statement

The animal study was reviewed and approved by Laval University Ethics Committee on animal research.

## Author contributions

CC carried out the experiments, analyzed the data, and wrote the manuscript.

## Funding

The author acknowledges the funding provided through grants by the Natural Sciences and Engineering Research Council of Canada (NSERC) as well as the Canadian Institutes of Health Research (CIHR).

## Conflict of interest

The author declares that the research was conducted in the absence of any commercial or financial relationships that could be construed as a potential conflict of interest.

## Publisher's note

All claims expressed in this article are solely those of the authors and do not necessarily represent those of their affiliated organizations, or those of the publisher, the editors and the reviewers. Any product that may be evaluated in this article, or claim that may be made by its manufacturer, is not guaranteed or endorsed by the publisher.

## Abbreviations

MCx: motor cortex
EMG: electromyographic
MT: motor threshold
ICMS: intracortical microstimulation
BIC: bicuculline.
